# Three decades of discovery: An overview of Hendra virus, the original Henipavirus

**DOI:** 10.1371/journal.pntd.0014138

**Published:** 2026-03-27

**Authors:** Kim Halpin, Raúl Gómez Román

**Affiliations:** 1 Australian Centre for Disease Preparedness, CSIRO, Geelong, Australia; 2 Independent Science Consultant, Ensenada, Mexico; Institute of Continuing Medical Education of Ioannina, GREECE

## Abstract

Hendra virus (HeV) emerged in Australia in 1994, causing a devastating outbreak among horses in Brisbane with spread to humans, resulting in one death. This nonsegmented, negative-stranded RNA virus belongs to the family *Paramyxoviridae* and represents the first zoonotic paramyxovirus isolated from bats. Flying foxes (genus *Pteropus*) serve as the natural reservoir, with all four mainland Australian species carrying antibodies with no apparent disease. HeV initiates infection by binding ephrin-B2 receptors on vascular endothelial cells, driving characteristic pathology involving vasculitis, thrombosis, and neurological complications. Horses are amplifying hosts, shedding virus abundantly in respiratory secretions and posing transmission risks to humans during invasive procedures. To date, seven confirmed human infections have been documented, with a 57% fatality rate, presenting as severe respiratory disease or progressive encephalitis. Two genetic variants are now recognized: the original HeV genotype 1 and the emerging HeV genotype 2, identified in limited equine cases. Recent surveillance of bat roosts revealed substantial viral diversity, with peak shedding occurring during winter—coinciding with equine spillover peaks. Prevention integrates multiple strategies: the licensed equine vaccine Equivac which provides One Health protection for both horses and human contacts; biosecurity measures including proper PPE; and habitat restoration to reduce nutritional stress in bat populations. Emerging therapeutics include monoclonal antibodies, with m102.4 showing cross-protective activity against both HeV and the closely related Nipah virus. No licensed human vaccines currently exist, though candidates are in development. Future prevention strategies increasingly recognize the importance of Indigenous-led conservation approaches alongside biomedical interventions. This review will focus on the history of HeV, virus replication and diversity, epidemiology, clinical manifestations, diagnosis, treatment, prevention, as well as ecological and interdisciplinary countermeasures.

## Author summary

Hendra virus (HeV) was first detected in 1994, with two outbreaks occurring within 2 months of that year. One was the index outbreak in the Brisbane suburb of Hendra, and the other was retrospectively diagnosed in the following year. This review examines the discoveries that have been made in the 30 years since its discovery.

## History

In early September 1994, a pregnant mare named Drama Series fell ill in a paddock on the southern side of the Brisbane River. Belonging to prominent horse trainer Vic Rail, she was transported to his racing stables in Hendra (~7 km from Brisbane’s center), which housed 23 other horses. Drama Series died dramatically escaping her stall, staggering outside, collapsing and rising repeatedly, crashing into obstacles throughout the night. The illness spread rapidly: 12 more horses became severely ill and died or were humanely euthanised, while 7 suffered mild illness before recovering, though they were subsequently euthanised under disease control protocols. Vic Rail also contracted the illness and died, though a stable hand recovered from what appeared to be a flu-like infection [1,2].

This was a zoonosis—a disease jumping from animals to humans—serving as a stark reminder that pathogens emerge from natural sources and can cross species boundaries without warning.

Initially, the equine disease resembled African Horse Sickness (AHS), an orbivirus from the *Reoviridae* family never before recorded in Australia. However, diagnostic testing at the Australian Animal Health Laboratory (AAHL, now the Australian Centre for Disease Preparedness; ACDP) ruled out AHS. Spleen and lung homogenates from two horses were inoculated onto Vero cell cultures. Cytopathic effect (CPE) was first observed on day 3 post‑inoculation in the cultures inoculated with lung homogenate [1]. At first, the CPE was thought to be caused by an equine herpes virus; however, electron microscopy showed the virus belonged to the family *Paramyxoviridae*. The causative agent was identified as a novel virus, initially termed equine morbillivirus (EMV), later renamed HeV when it became clear the virus was neither a morbillivirus nor a virus of horses [1].

The 1994 Hendra outbreak would not be the only one that year. Retrospectively diagnosed in 1995, the first recognized human HeV infection occurred when a man assisted his veterinarian wife in performing an autopsy on a horse that had died suddenly in a paddock in August 1994 [3]. After an initial brief illness, he relapsed 13 months later, developing fatal encephalitis [4].

## Description of the pathogen

HeV is a nonsegmented, negative-stranded RNA virus belonging to the family *Paramyxoviridae*, genus *Henipavirus*. HeV and Nipah virus (NiV) were the founding members of this genus. Several additional henipaviruses—namely Cedar virus, Angavokely virus, and Ghanaian bat henipavirus—have since been identified in Australia, Madagascar, and Ghana, respectively [5–7]. The HeV genome is unusually large for a paramyxovirus at 18,234 nucleotides, a feature partly attributable to extended untranslated regions (UTRs) at the 3′ ends of transcription units—a characteristic shared with filoviruses such as Ebola and Marburg [8,9]. Like other members of the *Orthoparamyxovirinae* subfamily, HeV adheres to the “rule of six,” whereby genome length must be a multiple of six nucleotides to replicate efficiently [10]. Each nucleoprotein subunit binds six nucleotides of genomic RNA, forming the template for transcription and replication.

The HeV genome encodes six structural proteins: nucleocapsid (N), phosphoprotein (P), matrix (M), fusion (F), attachment glycoprotein (G), and the large polymerase (L). Comparative sequencing across multiple horse outbreak isolates reveals remarkable genetic stability, with genomes sharing at least 99% similarity to the original 1994 strain [11].

Structurally, HeV resembles other paramyxoviruses: it is pleomorphic, enveloped, and contains a herringbone-like nucleocapsid. Virions range from 40 to 600 nm in diameter, noting the smallest versions are in filamentous form: G and F protein spikes protruding from the lipid envelope [12]. A distinguishing feature from its closest relative, NiV, is that HeV surface projections are typically double, whereas NiV’s are predominantly single.

### Genetic diversity

In 2021, retrospective investigation identified a second HeV variant, designated Hendra virus genotype 2 (HeV-g2), while the original strain is now called Hendra virus genotype 1 (HeV-g1) [13]. HeV-g2 has been detected in only two fatal horse cases (2015 and 2021) [14,15] and in flying foxes [13,16], suggesting it represents an emerging lineage with limited spillover history to date.

Recent surveillance has substantially expanded our understanding of HeV diversity. Researchers monitoring eight bat roosts over 3 years screened nearly 10,000 samples, identifying 629 positive for HeV [17]. Whole-genome sequencing revealed persistent viral RNA year-round in bats, with peak loads occurring in winter—precisely when equine spillovers peak (June, July, and August). Analysis of 48 bat HeV-g1 genomes and nine horse Hev-g1 genomes from historical outbreaks identified four main lineages plus additional cryptic variants. Notably, some bat strains matched those responsible for fatal horse and human infections, while others were entirely novel. These findings indicate that flying foxes, which travel long distances and roost in mixed-species groups, harbor substantially greater HeV diversity than previously recognized. Lineage-defining mutations predominantly occurred in the P/V/W and L proteins, with very few in the G glycoprotein [17].

### Viral entry and replication

The HeV replication cycle has been reviewed by others [18,19] and is summarized here briefly. HeV initiates infection by binding to ephrin-B2, a receptor widely expressed on neurons, smooth muscle, and endothelial cells lining small arteries ([20]. The viral attachment glycoprotein (G) engages the receptor, triggering activation of the fusion protein (F) with cleavage of F into two linked subunits (F1 and F2). The F protein mediates fusion between the viral envelope and host cell membrane, permitting entry of the viral ribonucleocapsid into the cytoplasm [8,21].

HeV replication occurs entirely in the cytoplasm and is mediated by the virally encoded RNA-dependent RNA polymerase complex composed of the L protein and its cofactor P. The nucleocapsid, consisting of genomic RNA encapsidated by N, serves as the template for sequential transcription of capped and polyadenylated viral mRNAs. As N protein accumulates, the polymerase shifts from transcription to genome replication, generating full-length antigenome intermediates and subsequently new genomic RNA, which are immediately encapsidated by N. The P gene additionally encodes accessory proteins (V, W, and C) through RNA editing and alternative open reading frames; these proteins antagonize type I interferon signaling and modulate host innate immune responses, thereby enhancing viral replication and pathogenicity. Viral assembly occurs at the plasma membrane, where the M protein coordinates interaction between the ribonucleoprotein complex and the envelope glycoproteins. Budding of mature virions is driven primarily by M protein–mediated membrane remodeling, completing the replication cycle [18,19].

## Epidemiology

### Reservoir host and spillover events

The natural reservoir is fruit bats, commonly referred to as flying foxes (genus *Pteropus*, family *Pteropodidae*). The identification of fruit bats as the reservoir of HeV marked the first reported isolation of a zoonotic paramyxovirus from bats [22]. To date, horses are the only domestic species to acquire direct infection from bats; they serve as amplifying hosts capable of transmitting the virus to humans and, very occasionally, other animal species.

Antibodies against HeV have been detected in all four flying fox species inhabiting mainland Australia, with the black flying fox (*Pteropus alecto*) and spectacled flying fox (*Pteropus conspicillatus*) identified as the primary contributors to spillover events in horses [23]. Antibodies to henipaviruses, including HeV have been found in other Pteropus species in Indonesia, Papua New Guinea, and Timor Leste [24–26]. Seroprevalence of HeV among flying fox populations varies considerably by location and season. In one monitored colony, seroprevalence increased steadily from 45% to 69% over 2 years, demonstrating endemic infection within that population [27].

Notably, HeV infection causes no apparent disease in fruit bats. Experimental studies confirm that infected Pteropus bats typically develop subclinical infections with only sporadic, low-level viral shedding in urine [28]. While some bats seroconvert and others show evidence of infection through viral genetic material in excretions (oral and rectal swabs, urine, and blood), viral antigens in tissues are rarely detected. This limited transmissibility among experimentally exposed fruit bats correlates with the infrequent occurrence of equine spillover events in the field [28].

### Transmission risk and temporal dynamics

In contrast, infected horses shed HeV abundantly in nasopharyngeal secretions even before clinical signs appear. Once disease becomes evident, viral replication extends throughout the horse’s blood, body fluids, and tissues [29]. Horses pose a transmission risk to humans beginning 72 hours prior to symptom onset and continuing through death and postmortem handling [29]. This risk intensifies as disease progresses, peaking at the time of death.

**Fig 1:**
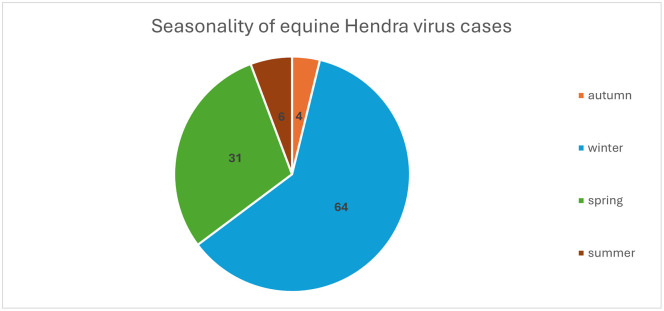
Seasonality of equine Hendra virus cases: numbers represent the number of equine cases per season; between August 1994 and February 2026, total number of cases is 105 [31].

**Fig 2:**
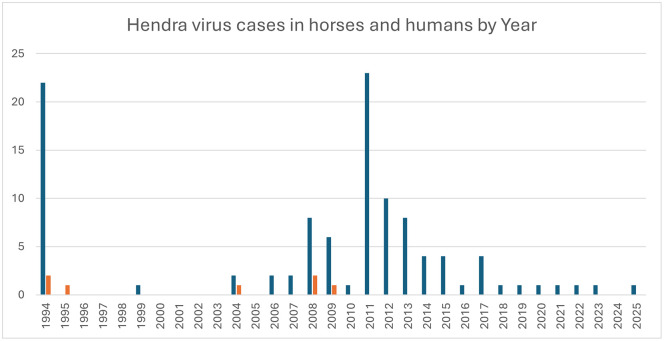
Number of horse (blue bars) and human (red bars) Hendra virus cases by year; between August 1994 and February 2026 total number of HeV infected horses is 105; total number of humans is 7 with 4 deaths and 3 survivors [31].

Spillover events peak in winter, followed by spring, corresponding with peak viral shedding from flying foxes (Fig 1) [30]. Horses become infected through direct contact with infectious body fluids (predominantly urine and sometimes fetal fluid) which contaminate pasture, feed, and water troughs. Since 2018, Australia has recorded approximately one equine case annually, with the notable exception of 2024, which saw no reported cases. Between August 1994 and February 2026, there have been 105 horses infected with HeV in Australia. (Fig 2) [31].

### Secondary host susceptibility

Among nonequine domestic species, susceptibility to HeV infection varies substantially. Dogs can acquire HeV infection but typically remain asymptomatic, developing antibodies without clinical manifestation—a pattern observed both in natural and experimental settings [32,33]. Similarly, pigs can be infected experimentally without displaying overt disease unless challenged with very high doses of virus [34]. In contrast, experimentally infected cats develop severe systemic vasculitis that is often fatal [35].

## Pathophysiology

HeV infection begins when the virus enters the host through inhalation or direct contact with infected bodily fluids (saliva, urine, and respiratory secretions). The viral attachment (G) and fusion (F) HeV glycoproteins mediate entry through interaction with ephrin-B2 receptors which are abundantly expressed on vascular endothelial cells and are also present on neurons and other central nervous system (CNS) cell types [20]. This receptor tropism underlies a defining feature of HeV disease: widespread endothelial infection leading to vasculitis, thrombosis, ischemia, necrosis, and CNS involvement [36]. Newly formed virions bud from infected cells and disseminate to other tissues, particularly the respiratory and CNS. In the lungs, viral-induced damage to the parenchyma causes respiratory symptoms including dyspnea and pneumonia [36]. CNS infection leads to neurological manifestations including headache, seizures, and encephalitis, with potential for long-term neurological sequelae [37].

Autopsy studies of fatal human HeV infection provide critical insight into the underlying mechanisms of disease. In one acute fatal case, viral antigen was widely detected in endothelial cells and parenchymal tissues of multiple organs, including lung, brain, and kidney, accompanied by vasculitis, thrombosis, and microinfarction [38]. These findings demonstrate that systemic microvascular injury is central to acute HeV pathogenesis. Notably, despite substantial cerebral vascular involvement, there was no clear ante-mortem clinical evidence of encephalitis in this patient [38], underscoring the contribution of vascular pathology to disease severity.

In contrast, a second fatal case demonstrated isolated viral encephalitis without inflammatory involvement of other organs [38]. Accumulating clinicopathologic evidence supports a dual pathogenetic mechanism in which HeV causes (i) systemic vasculopathy mediated by endothelial infection, resulting in thrombosis and ischemic injury, and (ii) direct neuronal and parenchymal infection within the CNS [39,40]. Experimental infection studies reinforce this framework; the hamster model of acute HeV infection reproduces both systemic vascular pathology and CNS involvement observed in human disease [41].

A critical aspect of HeV pathophysiology is its evasion of innate immunity. Henipavirus P gene-encoded proteins, including the V and W proteins, antagonize type I interferon signaling, a key antiviral defence mechanism, thereby permitting unchecked viral replication [42].

The occurrence of relapsing or late-onset encephalitis reported months after initial HeV infection (including one case occurring approximately 13 months after acute disease), and even years after NiV infection, suggests that viral clearance from the CNS may, in rare cases, be incomplete. One possible explanation is persistence within CNS tissue, potentially involving neurons or supporting glial cells, where reduced immune surveillance and continued antagonism of interferon-mediated antiviral signaling may contribute to incomplete viral clearance. Although direct evidence of long-term HeV persistence in human CNS tissue remains limited, persistence of NiV in the brains of nonhuman primates following apparent clinical recovery has been demonstrated [43], providing biologic plausibility for delayed neurologic manifestations across henipaviruses.

Together, these findings underscore that HeV disease severity reflects a complex interplay between endothelial tropism, viral immune evasion, and host inflammatory responses, which together shape both acute systemic disease and delayed neurologic complications.

## Clinical manifestation

### Clinical presentation in horses

Early reports characterized HeV infection in horses as acute respiratory syndrome, but subsequent outbreaks revealed a broader clinical spectrum including colic-like symptoms, sudden death, and neurological abnormalities [1,44]. The incubation period typically ranges from 4 to 16 days. Clinical signs include fever, tachycardia, anorexia, depression, dyspnea, and restlessness, sometimes accompanied by frothy or blood-tinged nasal discharge [29,45]. Some horses develop ataxia and myoclonus. Acute illness leading to death remains the most common presentation [44].

### Clinical presentation in humans

Initial human infections were characterized by severe respiratory disease, while subsequent fatalities involved relapsing or progressive encephalitis [1,4,37]. Among survivors, illness typically presented as acute influenza-like symptoms [46].

Of the seven confirmed human infections to date, four resulted in death (case fatality rate: 57%). Interesting cases include a veterinarian who conducted a horse autopsy and developed mild flu-like illness with recovery and persistent neutralizing antibodies 8 years post-exposure [47]; a veterinarian who performed nasal lavage on an infected horse and subsequently died [37]; a veterinary nurse assisting with that procedure who progressed from flu-like illness to acute encephalitis with persistent neurological sequelae [37,47]; and a veterinarian who performed endoscopy on an infected horse [48].

### Risk factors

The primary transmission risk occurs among individuals with direct contact to infected horse body fluids, particularly during invasive diagnostic or therapeutic procedures and when full personal protective equipment (PPE) is not utilized.

## Diagnosis

### Biosafety and sample handling

HeV is classified as a biosafety level 4 (BSL-4) pathogen, requiring the highest precautions for specimen collection and handling [45]. Both ante-mortem and post-mortem sampling must be performed under conditions preventing exposure of inadequately protected personnel to infected tissues or fluids. Full PPE, including biohazard respirators, is strongly recommended. Clinical suspicion should be raised when compatible syndromes occur in regions inhabited by the reservoir host (flying foxes). Some routine molecular and ELISA-based diagnostics can be performed at BSL-2 following validated inactivation of samples, provided appropriate upstream containment (BSL-3/4) is in place.

### Molecular assays

Real-time PCR amplifying the M and/or N or P genes is the gold standard for acute HeV infection. HeV can be detected in ante-mortem specimens (EDTA-anticoagulated blood, serum, oral, nasal and rectal swabs, cerebrospinal fluid, and urine) and post-mortem tissues (lung, spleen, kidney, tonsil, meninges, and lymph nodes).

### Virus isolation

The virus replicates readily in cell culture (Vero and RK13 cells), with cytopathic effects typically appearing within 2–3 days, characterized by extensive syncytial formation with peripheral nuclear localization in multinucleated giant cells. Suspect viral isolates are confirmed by molecular assays (PCR and/or sequencing). Immunofluorescence assays are not definitive due to sero-cross-reactivity among henipaviruses.

### Serological assays

The serum neutralization test (SNT) remains the reference standard for antibody detection and validation of alternative assays, but is confined to BSL-4 facilities due to the requirement for infectious virus propagation. Enzyme-linked immunosorbent assays (ELISA) utilizing soluble G protein serve as the preferred screening tool, particularly for surveillance applications. Positive ELISA results require SNT confirmation. A differentiating infected from vaccinated animals (DIVA) assay is available for equine populations. Serological surveillance is the method of choice for demonstrating absence of infection in animal populations.

Table 1 summarizes the main use cases for available HeV diagnostics and their application across horses, humans, and wildlife. In practice, suspected equine cases are investigated with real-time RT-PCR on EDTA whole blood, serum and pooled oral and/or nasal swabs as the first-line assay. Any positive results confirm infection and trigger assessment and testing of human contacts, whereas negative results in high-suspicion cases warrant repeat sampling and, if the animal dies, post-mortem tissue PCR and immunohistochemistry. Serology is useful for testing in-contact animals, conducting sero-epidemiological surveys and monitoring vaccine responses using DIVA assays, while analogous real-time RT-PCR and serological tools are applied to human contacts and to wildlife and environmental samples within a coherent One Health framework. To reduce dependence on BSL-4 facilities, surrogate neutralization assays using henipavirus glycoprotein-pseudotyped particles or competitive inhibition of receptor binding have been developed for use at BSL-2, but they still require calibration against the SNT. Prototype isothermal amplification assays (e.g., RT-LAMP) are being developed for near–point-of-care screening and may ultimately complement laboratory-based real-time RT-PCR [49].

**Table 1:** Summary of Hendra virus (HeV) diagnostic assays currently in use.

Purpose	Species (context)	Assay type	Target	Typical specimens	Biosafety level*	Turnaround time	References
Confirm acute clinical infection	Horses, humans	Real-time RT-PCR	M and/or N gene (±P/G)	EDTA whole blood; pooled nasal/oral swabs; other respiratory samples; CSF	Pre-inactivation: BSL-3/4; post-inactivation extraction and PCR: BSL-2	Same day (≤24 h)	[45,50,51]
Supplemental molecular confirmation/genotyping	Horses, bats, humans	Conventional/semi-nested RT-PCR with Sanger sequencing	M, P, N gene fragments	High-load clinical samples; tissues (lung, kidney, brain, lymph node)	Pre-inactivation: BSL-3/4; post-inactivation: BSL-2	1–3 days	[51,52]
Post-mortem confirmation in suspect cases	Horses (index or contact cases), experimentally infected animals	Immunohistochemistry (IHC) on FFPE tissue	HeV N or G antigen	Lung, brain/spinal cord, kidney, spleen, lymph node, uterus/placenta	BSL-2 (fixed tissue)	1–3 days	[45,53]
Definitive diagnosis/virus characterization	Horses, bats, humans (selected cases)	Virus isolation in cell culture (e.g., Vero, RK13) with Real-time RT-PCR confirmation	Whole virus; CPE (syncytia)	Same as for acute diagnostics or tissues; often high-load samples	Full procedure in BSL-4	5–10 days	[45,51]
Reference standard for neutralizing antibodies	Horses, humans, bats and other mammals	Serum neutralization test (SNT) with live HeV	Functional neutralization of infectious virus	Serum	BSL-4	3+ days	[51,52]
Screening for past exposure/infection	Horses, bats, humans	Indirect IgG ELISA using recombinant G glycoprotein	Anti-HeV G IgG	Serum	BSL-2	1 day	[52,54]
Screening for past exposure/infection	Bats, humans	Bead-based microsphere immuno-assay (Luminex)	Anti-HeV G IgG	Serum	BSL-2	1 day	[55]
Detection of recent infection	Horses	IgM capture ELISA (N-based or G-based formats)	Anti-HeV IgM	Serum	BSL-2	1 day	[56]
Differentiating infected from vaccinated animals (DIVA)	Vaccinated vs. naturally infected horses	Indirect/competitive ELISA using recombinant G and N antigens	Differential anti-G vs anti-N antibody patterns	Serum	BSL-2	1 day	[57]
Measurement of neutralizing antibodies at lower levels of containment	Horses, humans, experimental animals	Pseudovirus-based or surrogate neutralization assays (HeV/NiV glycoprotein-pseudotyped particles; receptor-blocking sVNT)	Inhibition of entry mediated by HeV/NiV F and G, or receptor binding	Serum	BSL-2	1–3 days	[58,59]
Eco-epidemiological and reservoir investigations	Pteropid bats, other wildlife, environmental samples	Real-time RT-PCR; NGS, ELISA, neutralization assays	M/N gene; anti-HeV antibodies	Bat urine collected under colonies, oral/rectal swabs, tissues; environmental swabs; serum	Pre-inactivation: BSL-3/4; post-inactivation: BSL-2	Variable (1–7 days)	[13,16,17,45,51]

* Biosafety levels are indicative and may vary according to national regulations and institutional risk assessments.

## Treatment

There are currently no licensed therapeutics to treat HeV infection in humans or animals. Management of confirmed or suspected cases, therefore, relies on intensive supportive care, timely recognition of complications (respiratory failure, encephalitis, and multi-organ dysfunction) and strict implementation of infection prevention and control measures. In horses, clinical management is constrained by regulatory requirements and occupational health considerations; in most Australian jurisdictions, humane euthanasia of confirmed or strongly suspected equine cases is recommended to protect veterinary staff and prevent onward zoonotic transmission [22].

An overview of emerging medical countermeasures for henipaviruses, including monoclonal antibodies (mAbs), small-molecule antivirals, and vaccines, has been provided by Gómez Román and colleagues [60]. Among specific therapeutics, monoclonal antibodies targeting the viral attachment (G or receptor-binding protein [RBP]) and fusion (F) glycoproteins are the most advanced candidates. The human mAb m102.4, directed against the henipavirus RBP, has shown potent cross-neutralizing activity against both HeV and NiV viruses and conferred complete protection in multiple animal models when administered shortly after exposure ([61–63]; reviewed in [60]). A phase I trial in healthy adults demonstrated an acceptable safety profile for single and repeated intravenous doses, and m102.4 has been used under compassionate-use protocols as post-exposure prophylaxis in individuals with high-risk exposures to infected horses, although efficacy in humans has not yet been formally established [64–66].

Complementing G-directed antibodies, several F-targeting mAbs have been developed. The humanized antibody h5B3.1 recognizes a prefusion-specific quaternary epitope on the F glycoprotein that is conserved between NiV and HeV, potently neutralizing both and blocking membrane fusion in vitro [66]. In ferret models of lethal NiV and HeV challenge, post-exposure administration of h5B3.1 at 24–72 hours after infection conferred robust protection, representing the first demonstration of successful post-exposure therapy with an F-directed humanized mAb against henipaviruses [64]. These data support evaluation of combination regimens that jointly target F and G/RBP.

Naturally occurring human mAbs isolated from vaccinated or convalescent donors further expand the therapeutic landscape. Dong et al. identified a panel of HeV-RBP-specific antibodies that map to at least five distinct antigenic sites, including cross-reactive mAbs HENV-26 and HENV-32, which afford post-exposure protection in ferrets challenged with NiV [67]. Structural studies reveal diverse mechanisms of neutralization, including direct competition with receptor binding and allosteric inhibition of conformational changes required for fusion [67]. These findings highlight multiple, nonoverlapping sites of vulnerability on the henipavirus RBP that can be exploited for rational antibody cocktail design.

The emergence of the divergent HeV genotype 2 (HeV-g2) has prompted specific efforts to ensure that candidate biologics retain activity against this lineage. Wang *et al*. reported that several previously described HeV-neutralizing antibodies cross-neutralize HeV-g2, and that a murine mAb, hAH1.3, targeting the G/RBP head domain can be combined with other nonoverlapping antibodies to achieve potent neutralization of both prototype HeV-g1 and HeV-g2 [68]. These data support the development of multivalent antibody cocktails to optimize treatment efficacy as henipaviruses diversify in reservoir bat populations.

More recently, Isaacs and colleagues described a bispecific antibody that directly addresses the risk of viral escape. A nanobody (DS90) targeting a conserved site on F was fused to an anti-RBP mAb (m102.4), generating a dual-specific molecule that neutralizes HeV and NiV, prevents the selection of escape mutants in vitro and provides superior protection from NiV disease in animal models compared with monotherapy [69]. This bispecific approach exemplifies how combining F- and G/RBP-directed activities in a single construct may increase potency and the genetic barrier to resistance.

## Prevention

Prevention of HeV spillover events and further transmission should aim to integrate nonpharmaceutical (biosecurity) and pharmaceutical (vaccines) countermeasures.

In terms of biosecurity countermeasures, using effective PPE when interacting with a sick horse is vital to protecting those around the sick horse. There are many instructional videos and information online to help horse owners and veterinarians in the field working with horses [70].

HeV does not persist well outside its host, as it is highly sensitive to drying and temperature fluctuations. Depending on conditions, it may remain viable in the environment for only a few hours to several days; for precautionary purposes, a maximum survival of 5 days is assumed under ideal conditions. Effective inactivation can be achieved with common disinfectants, including soap and detergents, Virkon, hypochlorites, iodophors/iodine, biguanidines such as chlorhexidine, and quaternary ammonium compounds.

The control measures in outbreaks of HeV disease are governed by its extreme hazard as a zoonotic agent. It is essential to prevent spread of infection among horses, and to preclude the possibility of infection of humans. Rapid eradication is recommended. This is achieved on a regular basis in Australia by the quarantine of infected premises and the isolation of infected horses, and testing of all horses on the property. It is advisable to keep dogs and cats away from infected animals until quarantine orders are lifted.

Simple biosecurity measures can protect horses. Fruit trees and other vegetation that may attract fruit bats should be removed from the proximity of feeding and water troughs and stables. Feeding and water troughs should be covered at night if in an endemic area, as bats are more active during the night [71].

In terms of pharmaceutical countermeasures, there is a highly effective HeV horse vaccine. Equivac HeV vaccine (Zoetis) was released in 2012 and in 2015 became fully licensed. The vaccine uses a soluble G antigen which is the HeV attachment glycoprotein on the surface of the virus. This vaccine is described as a One Health vaccine because vaccinating horses not only protects them from infection but also protects the people who interact with the horses, effectively removing the need for a separate human vaccine [72].

Monitoring of the recommended annual vaccination schedules through accurate record-keeping is made easy by an online vaccine registry managed by the vaccine manufacturer. Horses must be microchipped, and the microchip is scanned and recorded at all consultations. Veterinarians can check records of new patients to ensure vaccine coverage is maintained. Likewise, if a horse becomes ill, the registry can be used to check vaccine status. A large study has shown that provided horses received at least three vaccinations (consisting of two doses 3–6 weeks apart, and a third dose 6 months later), horses had high neutralizing titers (median titer for three or more vaccinations was 2,048), and none tested negative [73].

For humans, there are no currently licensed HeV vaccines, though there are several vaccines in the pipeline (reviewed in [60] and [74]). It is worth noting that one of the henipavirus human vaccine candidates is a vaccine for NiV which uses the same antigen that is used in the Equivac HeV horse vaccine [75]. In addition, new data were presented during the Hendra@30 Conference [76]. This included: pre-clinical and Phase I clinical data for mRNA-1215, with early pre-clinical evidence on cross protection against NiV and HeV infections; the prospect of Polyphosphazene (PPZ) adjuvanted micro-needle patch (MNP) vaccines based on the HeV protein subunit platform; and several pre-clinical studies in animal models with good evidence of cross protection against HeV and NiV. Others have also discussed the prospect of pan-henipavirus vaccines, possibly incorporating components that elicit broad cross protection against several henipaviruses.

## Ecological and interdisciplinary countermeasures

In addition to pharmaceutical and nonpharmaceutical countermeasures, prevention should include the active preservation and restoration of flying fox habitat to reduce viral shedding. Flying foxes prefer native forests rather than suburban and peri-urban parks. Bayesian analyses of long-term HeV datasets from eastern Australia show that spillover risk surges when bats are nutritionally stressed and foraging in agricultural landscapes, and collapses in years when abundant winter-flowering in remnant forests draws them away from horse paddocks; in short, well fed bats in healthy forests appear to be low risk neighbors for horses [76,77].

Against that backdrop, older Turrbal, Jagera, and Yuggera relationships with flying foxes around Brisbane take on a different resonance. Historical, secondhand sources suggest that flying foxes were a valued food within a broader cosmology that granted bats ancestral significance [78], and that use was small scale, without industrial culling, in a landscape without horses and before extensive clearing of winter-flowering trees [79]. Large roosts on Brisbane River islands, most notably St Helena and Indooroopilly, are documented both in historical accounts and in contemporary management records [78–81]. Nothing here should be interpreted as endorsing bat hunting today. On the contrary, the evidence points to bat conservation and habitat restoration as central to prevention. The apparent absence of a Hendra-like footprint in pre-colonial history invites Indigenous-led, interdisciplinary research on practices that sustained bat populations and habitats [82]. Future HeV policy may have as much to learn from Indigenous stewardship of Country as from virology and immunology.
